# French Surgery in the Crimea

**Published:** 1861-09

**Authors:** 


The general practice of the French Surgeons in the Crimea was to extract foreign bodies from wounds at an early period, whenever they were easily accessible. The most efficient styptics in arresting hemorrhage, where the blood-vessels could not be conveniently tied, were the perchloride and the persulphate of iron. Amputations were generally resorted to in severe injuries of the limbs, and the results were more favorable than when conservative surgery was attempted. Primary amputations were much more successful than secondary.

